# Supplementary material for: Acceptance and compliance with micronutrient powder (MNP) among children aged 6–23 months in northern Nigeria
Source: PLOS Glob Public Health. 2022 Oct 17;2(10):e0000961. doi: 10.1371/journal.pgph.0000961 (PMC10022258; doi:10.1371/journal.pgph.0000961)
Supplement: S1 Dataset — (ZIP) [file pgph.0000961.s008.zip › S8_Dataset/Spot Checks.zip!/Spot Checks/03072015_A_SO_PM_2_SC.pdf]

SPOT CHECK RECORDING FORM

State: _ADAMAWA_______________ LGA: SONG_________________ Community: BONGO_____________________
NAME OF CAREGIVER(FEMALE)………TALATU BABA GARIN

Sex of index child: _MALE_____________ Age of index child (in months): 23__________________

Spot Check Information

1. **Date of MNP distribution to household** (circle option below based on LGA name above)**:**
   - **Kebbi:** BirninKebbi&Jega LGA: **June 16**
   - **Kebbi:** Danko-wasagu: **June 17**
   - **Adamawa:**Song, Ganye: (/"June 18")
   - **Adamawa:**Guyuk **June 19**
2. **Date of Spot Check Observation: _1-07-2015__________________**
3. **Number of sachets remaining**(Ask the caregiver to show you and count the specific number of sachets remaining)**:**

   46

4. **Number of sachets that <u>should remain</u>using one MNP sachet per day** (Analyst only)**:**

   48

5. **Number of sachets remaining / number of sachets should be remaining**(Analyst only)**:**

____46_____ / ____48_____ = _____95.8____ %

Probes

Question 1. You have **XX** MNP sachets remaining. Explain how easy or difficult it was to use the MNP product on a daily basis for your child 6 – 23 months? (Circle one answer)

Very Difficult **Difficult** **(Easy)** **Very Easy**

Question 2. Describe any specific challenges that you faced that made the MNP difficult to use on a daily basis?

Question 3. Describe specific aspects of the MNP product that you or your child enjoyed that made it easier to use on a daily basis? IT IS TASTELESS.PROPORTIONATE QUANTITY.IT WILL DEVELOP THE BRAIN OF THE CHILD.IT DRIVES AWAY MINOR ILLNESSES.

Question 4. What questions do you have about the MNP based on your experience using it?
I WAS TOLD THE MNP HELPS DEVELOPS THE BRAIN OF A CHILD TO BE VERY INTELLIGENT,ASIDE THIS,DOES THE MNP HAVE ANY OTHER BENEFITS TO THE CHILD.? SECONDLY,CAN THE MNP BE MIXED WITH ANY OTHER FORM OF FOOD ASIDE SORGHUM GRUEL?

Question 5. What specific recommendations do you have that we can do as a program to make the MNP easier to use on a daily basis? THEY SHOULD PROVIDE READY TO USE FOOD TO BE MIXED WITH THE MNP.THEY SHOULD MAKE THE TASTE(MNP) VERY SWEET.
